# Developing 3D-Printable Cathode Electrode for Monolithically Printed Microbial Fuel Cells (MFCs)

**DOI:** 10.3390/molecules25163635

**Published:** 2020-08-10

**Authors:** Pavlina Theodosiou, John Greenman, Ioannis A. Ieropoulos

**Affiliations:** 1 Bristol Bioenergy Centre, Bristol Robotics Laboratory, University of the West of England, Bristol BS16 1QY, UK; john.greenman@uwe.ac.uk; 2 Department of Applied Sciences, University of the West of England, Bristol BS16 1QY, UK

**Keywords:** 3D-printing, electrode materials, alginate, air-breathing cathode, EVOBOT, MFC, additive manufacturing

## Abstract

Microbial Fuel Cells (MFCs) employ microbial electroactive species to convert chemical energy stored in organic matter, into electricity. The properties of MFCs have made the technology attractive for bioenergy production. However, a challenge to the mass production of MFCs is the time-consuming assembly process, which could perhaps be overcome using additive manufacturing (AM) processes. AM or 3D-printing has played an increasingly important role in advancing MFC technology, by substituting essential structural components with 3D-printed parts. This was precisely the line of work in the EVOBLISS project, which investigated materials that can be extruded from the EVOBOT platform for a monolithically printed MFC. The development of such inexpensive, eco-friendly, printable electrode material is described below. The electrode in examination (PTFE_FREE_AC), is a cathode made of alginate and activated carbon, and was tested against an off-the-shelf sintered carbon (AC_BLOCK) and a widely used activated carbon electrode (PTFE_AC). The results showed that the MFCs using PTFE_FREE_AC cathodes performed better compared to the PTFE_AC or AC_BLOCK, producing maximum power levels of 286 μW, 98 μW and 85 μW, respectively. In conclusion, this experiment demonstrated the development of an air-dried, extrudable (3D-printed) electrode material successfully incorporated in an MFC system and acting as a cathode electrode.

## 1. Introduction

Microbial Fuel Cells (MFCs) are bio-electrochemical transducers that use the metabolic activity of microorganisms to convert chemical energy (stored in organic matter) into electricity [1]. Typically, they have a biotic anode and an abiotic cathode, separated by a semi-permeable membrane. MFCs can utilise a vast collection of organic compounds, including organic waste, due to the diverse metabolism of the bacterial communities within the biotic anode [2]. This characteristic has led to a rapidly expanding international interest around the subject of MFCs for their ability to treat wastewater and harvest green energy [3]. Even though MFC research interest has bloomed over the past 20 years [4], it was, in fact, more than 100 years ago when the fundamental MFC concept was first reported by Potter [5]. 

Another example of a technology that had sparked interest over the last few decades is 3D printing technology (or additive manufacturing (AM)) which was first reported in 1983 by C. Hull [6]. Even though, at first, these two technologies may have appeared to have no relation at all, in reality, MFC and 3D-printing started co-existing in the literature in 2010 [7]. Since then, AM or 3D-printing are playing an increasingly important role in advancing MFC technology, by substituting essential structural components i.e., chassis and separators-with 3D-printed parts. This helped overcome many time-consuming MFC assembly steps and allowed the creation of intricate designs [8,9,10,11,12,13,14].

Recently, through the EVOBLISS project [15], the synergy between a 3D-printers and MFCs was investigated further. The project aimed to create an open source expandable and customisable robotic workstation (called EvoBot), to develop new materials and applications based on real-time feedback between the robot and the MFCs in an attempt to enhance MFC technology. Through EvoBot as the main apparatus, EVOBLISS took 3D printing technology to the next level by turning an open-source low-cost 3D printer to an interactive research tool that enabled the empirical study of adaptation and evolution of the MFC systems [16,17,18]. EvoBot is a modified 3D printer, so naturally, the ability to also extrude material for MFC parts, such as membranes [18] or electrodes, was explored. The main focus was using EvoBot both as a “maintenance machine” and a fabrication tool which can 3D-print core MFC materials, such as electrodes and membranes. This approach can lead to the production of optimised MFC reactors much faster than conventional methods and allow automated lab testing, before being employed in real world applications.

The study presented here focuses on identifying electrodes that can be 3D-printed and used directly on MFCs. This would be a big step towards advancing the technology for the mass manufacturing of MFCs using only 3D-printers. Thus, the line of experiments presented in this article focuses on identifying electrode materials that can be used within monolithically 3D-printed MFCs. This paper aims to describe the development of an inexpensive, conductive, eco-friendly and printable alginate–based electrode, which can be extruded from the EvoBot platform and report on the advances of this material as a cathode electrode on air-breathing cathodes.

## 2. Results

### 2.1. Continuous Power Output

**Figure 1:**
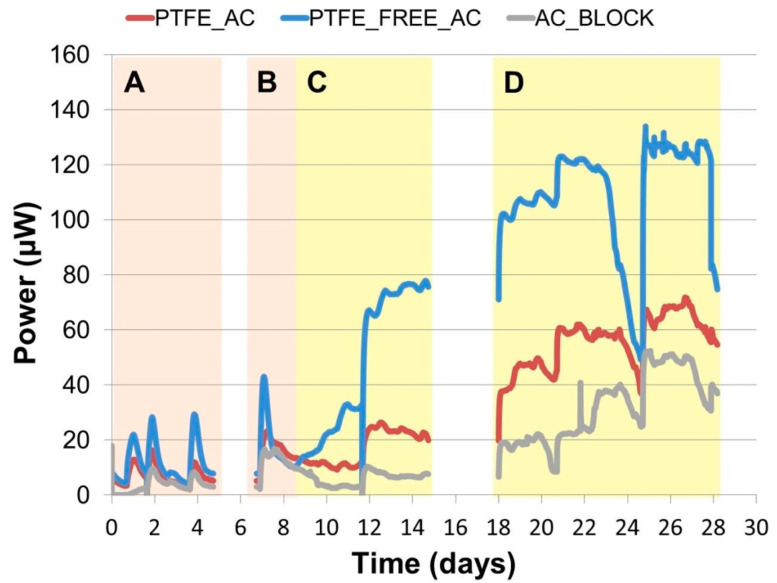
Real time power output of the MFCs employing different cathode electrodes. Different phases are annotated. (**A**) Inoculation phase consisting of three sludge exchanges as indicated from the spikes in the graph; (**B**) Single re-inoculation following a 48 h of power cut and loss of data; (**C**) Initiation of urine feeding cycle; (**D**) Continuation of urine feeding cycle following a 79 h period without monitoring.

At the initial stages of the experiment, the MFC triplicates were inoculated with tryptone and yeast extract (TYE)-supplemented activated sludge. The power output was increasing after each addition, as shown in Figure 1A,B. In all cases the MFCs almost doubled in power output from their initial sludge exchange until their fourth and last exchange that occurred on day 7. Initially PTFE_FREE_AC was performing at 21 μW, PTFE_AC at 14 μW and AC_BLOCK at 7 μW whereas on day 7 performance levels were at 42, 23 and 15 μW, respectively.

The end of the inoculation period signalled the beginning of the urine feeding cycle when initially 50% of the anolyte was removed and exchanged with neat urine (day 8), as shown in Figure 1C. At the beginning, this had a positive effect on the PTFE_FREE_AC MFCs, which saw an increase of 68.75% in power output; urine did not have the same effect on the other two MFCs that remained almost unaffected. To investigate this further, all the MFC anodes were emptied completely with the anolyte entirely removed even from the carbon veil electrode, and then replenished with 100% neat urine. The MFCs responded uniformly to this feeding and instantly the power output reached 67 μW for the PTFE_FREE_AC, 24 μW for the PTFE_AC and 10 μW for the AC_BLOCK. During the next 13 days, the MFCs steadily increased in power output despite a 79-h period during which they were left unattended. This shows the resilience of the system, which even after being left unattended for days, can continue to thrive as long as a carbon energy source is added to the system. This is shown more clearly from day 23 to day 25 when the MFCs steadily degraded their organic fuel but were reinvigorated when fed again (Figure 1D).

**Figure 2:**
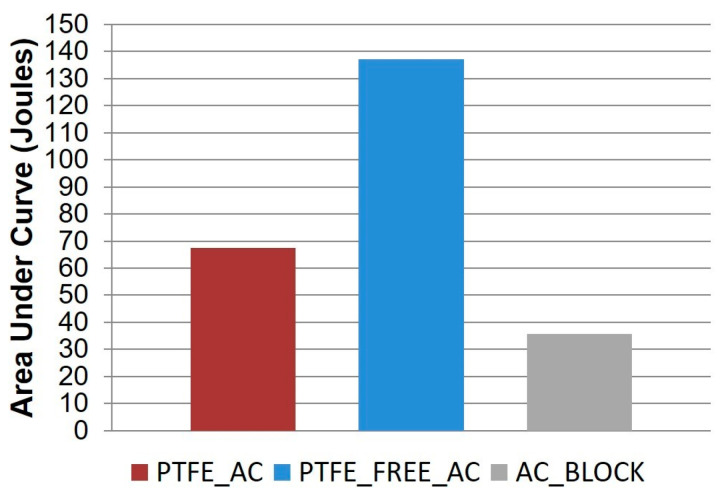
Overall calculated area under the curve, presented in Joules, based on the temporal power output data.

Looking at the temporal behaviour, the MFCs with the 3D-printed alginate-based cathode electrode (PTFE_FREE_AC) performed better than the control (PTFE_AC) and the commercially available sintered carbon block (AC_BLOCK) gave the lowest performance. The area under curve, which represents the total joules produced, was calculated for the whole experiment and is presented in Figure 2.

### 2.2. Polarisation Results

The polarisation experiment is an electrochemical analysis performed on MFCs by gradually sweeping the external resistance value starting from open circuit (high resistance value) and finishing with a very low resistance value. This experiment provides information on the power output capabilities of the MFC system by identifying the maximum power transfer point (MPT) that an MFC can achieve.

**Figure 3:**
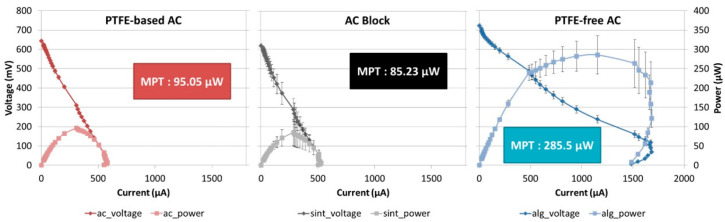
Polarisation results of the three types of MFCs examined. (n = 3).

Following the initial maturing stages, a polarisation experiment was conducted to identify the maximum power output capabilities of the three MFC systems. The results of this experiment are presented in Figure 3 and are in agreement in terms of the order of performance with the real-time (Figure 1) data. The MPT point for the MFC employing the alginate-based PTFE_FREE_AC electrode was 285.5 μW, which was 190.45 μW higher than the control PTFE_AC and 200.27 μW more than the sintered carbon block. The differences in power output magnitude between the real-time data and the polarisation data are due to Rext, which was not optimal during the experiment. The same constant Rext was intentionally employed for all MFCs, even though optimal Rext values for each cell would have also been appropriate; this was in order to avoid adding yet another variable in this comparative study. 

### 2.3. Cathode Electrode Lineal Sweep Voltammetry (LSV)

**Figure 4:**
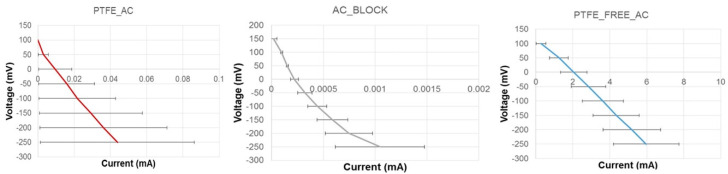
Linear sweep voltammetry results of the three cathode electrodes (250 mV/S) (n = 3).

To investigate the electrocatalytic activity of the air-breathing cathodes, LSV was carried out under clean conditions, using neat urine (pH = 9.20) as the electrolyte solution. Prior to running the LSV, the cells were left to soak overnight in the urine solution, ensuring liquid continuity between the membrane and the electrodes as well as achieving a stable open circuit potential (OCP) for all the cathodes tested. The resulting OCPs of the two custom made electrodes, PTFE_AC and PTFE_FREE_AC, were 85 ± 18 mV (vs. Ag/AgCl) and 108 ± 8 mV (vs. Ag/AgCl), respectively, whilst the commercially available AC_BLOCK had an OCP of around 163 ± 15 mV (vs. Ag/AgCl), which was the highest of all (Figure 4). This can be attributed to two factors. Firstly the AC carbon base employs granular activated carbon (GAC) instead of powdered activated carbon (PAC) and GAC has higher reactivity with gases than PAC. Secondly, during the manufacturing process, GAC is sintered into rigid blocks which increases the number of pores within the block, leaving a higher surface area for oxygen reduction compared to the other two tested electrodes, which were made manually with PAC.

All the electrodes were below the theoretical value of ORR vs. Ag/Ag Cl at pH = 9 which is 580 mV, primarily because these values are based on platinum electrodes and not carbon-based electrodes. Furthermore, OCP only shows thermodynamic differences in potentials between the anode and the cathode electrodes (against a reference electrode) which is not a determining factor of the performance of the MFC once the circuit is closed, as reflected from the power curves (Figure 3). The LSV data (Figure 4) show that the PTFE_AC and AC_BLOCK had low current outputs and also underwent higher activation losses than PTFE_FREE_AC. Overall, the LSV data confirm that the 3D-printed electrodes (PTFE_FREE_AC) produced notably higher current output than the rest (6 ± 1.5 mA at −250mV vs. Ag/AgCl).

## 3. Discussion

The results presented above confirmed that the PTFE_FREE_AC is superior to the other two electrodes, PTFE_AC and AC_BLOCK, in terms of power output during MFC real time operation and polarisation conditions. Additionally, PTFE_FREE_AC produced considerably higher current output levels than the rest during electrochemical examination. All three system configurations showed equal resilience during carbon energy depletion, which was reflected on their recovery profiles once fed. Notably, throughout the experiment, the total joules produced by PTFE_FREE_AC MFCs were more than double compared to the PTFE_AC MFCs and four times as much compared to the MFCs with AC_BLOCK cathodes. Power output is the ultimate parameter for determining the suitability of the MFCs to be used in practical applications. However, for making these systems accessible, affordable, and eco-friendly there are other factors that need to be taken into account, as well when designing and manufacturing MFCs. These considerations are the availability and usability of the materials, costs, and their ability to be used in 3D printers, such as EvoBot. All these are discussed in detail below.

### 3.1. Material Selection Rationale

#### 3.1.1. Activated Carbon

Activated carbon (AC) is a porous, solid surface material which can merge with different molecular structures [19]. In this experiment AC was used in its powder form (PAC) in the PTFE_AC and PTFE_FREE_AC electrode. AC derives from a wide range of carbon-rich raw materials, such as coconut shells and wood and it is activated using chemical or steam activation, of which the latter is more common. AC exhibits an extended inter-particulate surface area which offers to the material a high degree of porosity and excellent adsorbent characteristics [19]. This is attributed to its unique properties, such as: large surface area (500–2000 m^2^ aggregated surface area per gram of AC), high degree of surface reactivity, universal adsorption, and pore size (micro- and macro-pores), which allows adsorbents to easily attach to the inner surface of the AC. AC is the conductive element of choice when it comes to electrode materials for the MFCs of this study due to its availability, cost effectiveness and eco-friendliness.

#### 3.1.2. Sodium Alginate 

Sodium alginate was employed in this experiment as a binder for the PTFE_FREE_AC. As a material, sodium alginate is a versatile, economical, and high-modulus polysaccharide extracted from brown algae. Recently it has attracted a lot of interest as an eco-friendly binder for both anodic and cathodic electrodes in the lithium ion (Li-ion) battery industry [20,21,22,23]. The properties of alginate have been discussed in the literature in relation to using alginate as a printable carbon energy feedstock for MFCs [24]. In this experiment, the focus was to investigate whether it could be used as a biodegradable binder in MFC electrodes in an attempt to move away from employing the toxic PTFE binder. Similar to preparing a Li-ion battery electrode, the PTFE_FREE_AC alginate-based electrode was formed into a slurry by mixing the conductive carbon powder (PAC) with alginate as the polymeric binder and deionised water as a solvent. However, instead of casting the slurry into a metal foil current collector (as in Li-ion), it was deposited on the membrane directly using a syringe. 

Although binders are electrochemically inactive, they still play an important part in the stability and integrity of the electrodes [22] as well as the performance of the cell [20]. Additionally, water-processed binders, such as alginate, can air-cure and do not require further treatment. Besides, they are an economical and eco-friendly alternative to toxic electrode components and costly manufacturing processes.

#### 3.1.3. Carbon Block

Bonded activated carbon filters are used in a wide variety of purification techniques, including water purification, gas purification, and air filters. For such applications, granular activated carbon (GAC) is the common choice because it has the flexibility to be incorporated as a loose filler within filter housing or into carbon blocks [25]. The industrial manufacturing of bonded carbon filters requires the incorporation of various chemical processes in order to bond the carbon particles into a rigid matrix. Carbon block was used as a cathode electrode in the MFC set-up named AC_BLOCK.

Gas molecules are adsorbed into the carbon block by diffusing into the pores of AC where they get trapped in the walls. Since the surface area of the block is extremely large, the number of pores within the block is equally large. With these properties in mind, this type of filter was selected as the commercially available product to be trialled as the of-the-shelf air-breathing cathode electrode for the MFCs. In this study, the AC block was made out of coconut-based AC, which is considered effective as it offers high porosity and surface area, facilitating quick adsorption, which is why it is preferred for use in gas and water filters. Furthermore, coconut shells are found freely in nature, making the manufacturing process more sustainable.

The manufacturing process of bonded carbon filters from loose GAC proved to be disadvantageous for their use as air-breathing cathode electrodes in this study. This is because the chemical bonding process alters the carbon particles, having a detrimental effect on their ability to bond with gas compounds [19]. The bonding process initially requires the AC to be soaked in water for 24 h, which can cause the leaching of useful impregnated chemicals, followed by a soaking in bonding agents, such as polystyrene, which can affect the adsorption capacity of the granules. It is hypothesised that these factors influenced the underperformance of this type of electrode used herewith. Similar observations were published in a recent study, where sintered AC blocks were employed in membrane-less MFCs treating urine [26]. Among the different cathodes tested in that study, the AC block was the least performing but was the most economical, as presented in the cost analysis below.

### 3.2. Cost Analysis

An important factor to consider in the effort to improve MFC performance is not only the power output but also the cost effectiveness of the materials, especially when using alginate. In perspective, with the aim to reach an MFC value of GBP 1 per unit (less than the value of a rechargeable AA battery), the alginate electrode is the most suitable in reducing the overall costs. The PTFE_AC cathode electrode requires a PTFE coated carbon veil sheet as well as a mixture of PTFE and AC. PTFE is a highly toxic and expensive material (GBP 138/500 mL) compared to food grade alginate, which is 15-times cheaper at GBP 8.76 per 500 g. The assembly and manufacturing costs of PTFE_FREE_AC is even less that the conventional electrode (PTFE_AC), not only because of the binder, but because it does not require a supporting material or heat treatment to set. Hence, by removing the extra costs regarding materials and assembly for the cathode electrode using the 3D-printed ones, the cost per electrode (20 cm^2^) comes to GBP 0.035 each (not factoring in 3D-printing energy costs).

### 3.3. 3D-Printing Feasibility Using EVOBOT

**Figure 5:**
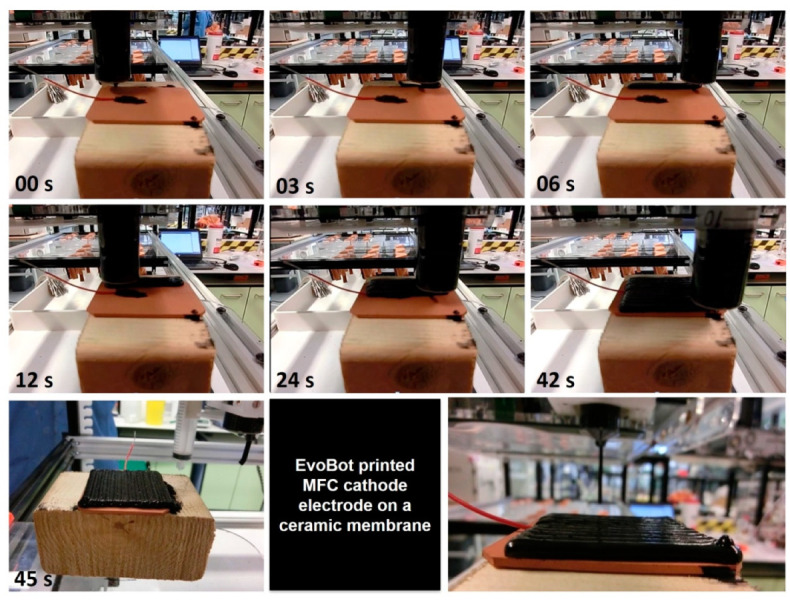
Time-lapse photographs of the EvoBot extrusion of the PTFE-free/alginate-based cathode electrode on a ceramic MFC membrane in less than a minute.

Following the successful implementation of the PTFE_FREE alginate-based electrode on MFC membranes using manual syringe extrusion, the next step was to test the same extrusion using syringes on the EvoBot [16]. For this test, the syringe was preloaded with conductive paste and attached to the robotic head without any modifications (i.e., implementing an extruder) because the viscosity of the paste was sufficient for the syringe motors to push the paste through. To extrude the conductive paste, the desired shape was created in CAD and the membrane was placed on the platform in a pre-set position. CAD dictated the path that the syringe nozzle of EvoBot needed to follow. A time-lapse figure of the EvoBot extruding the cathode electrode is shown below (Figure 5).

## 4. Materials and Methods 

### 4.1. MFC Architecture

**Figure 6:**
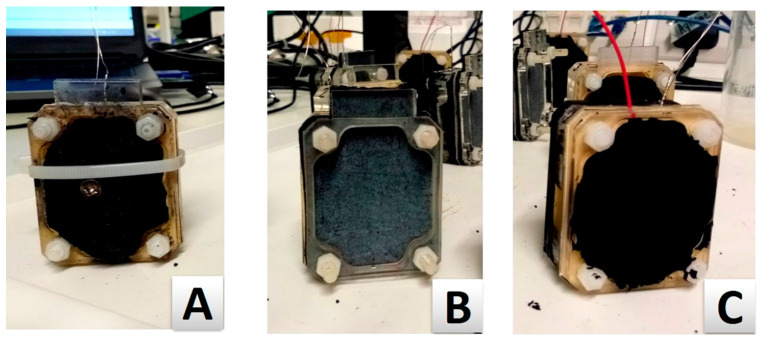
Photographs of the cathode electrodes used in this study. (**A**) AC_BLOCK: Activated carbon block as the commercial option, pierced through with a SS screw as the current collector; (**B**) PTFE_AC: Activated carbon on carbon veil, this PTFE-based electrode was used as the control; (**C**) PTFE_FREE_AC: PTFE-free alginate based electrode as the extrudable electrode.

Three triplicates of single chamber analytical cuboid type MFCs were assembled for this experiment as previously described [24]. The capacity of the anodic chamber was 25 mL. The cathodic half-cell was removed completely and the cathode electrodes in examination were directly attached to the CMI-7000S cation exchange membrane (CEM) (Membranes International, Ringwood, NJ, USA), forming open-to-air cathodes (Figure 6). The CEMs were cut to shape based on the MFC dimensions and then immersed in 5% NaCl solution for 12 h at 40 °C to allow hydration and expansion. The anode electrode used in all the MFCs was untreated (catalyst-free) carbon veil fibre, with 20 g/m^2^ carbon loading (PMF Composites, Dorset, UK). Each anode electrode had a surface area of 270 cm^2^.

### 4.2. Cathode Electrode Material

**Figure 7:**
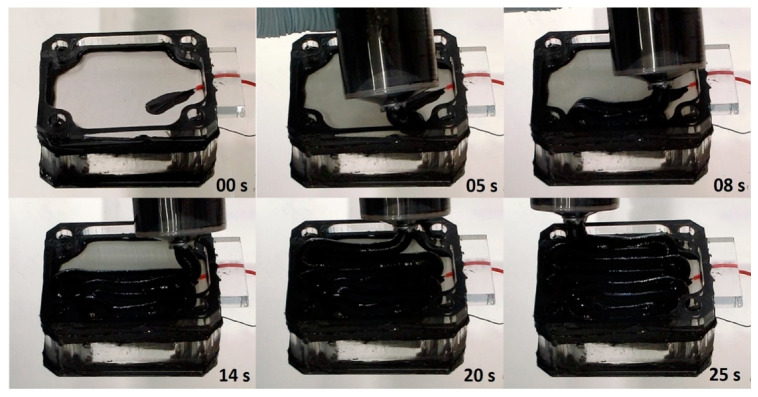
Time-lapse photography of the manual extrusion of the PTFE-free/alginate-based cathode electrode on the membrane of an assembled MFC.

**Figure 8:**
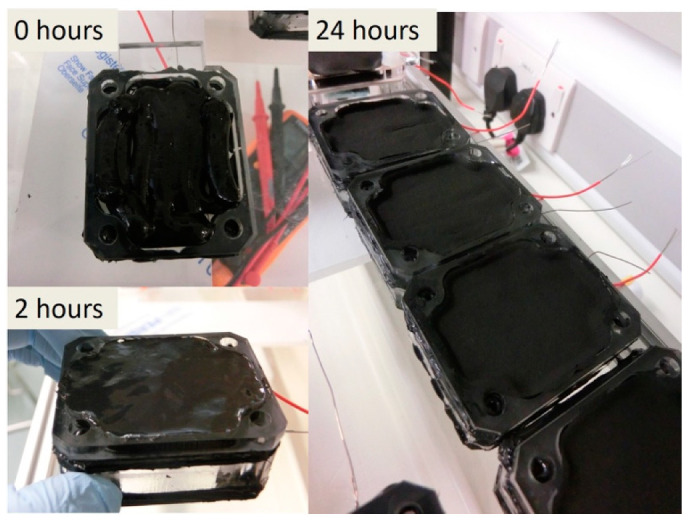
Manually extruded PTFE-free alginate electrode 0, 2 and 24 h after extrusion, showing the electrode settling and solidifying (air-drying).

Two types of cathode electrode material were tested against the control PTFE-based cathode electrode named PTFE_AC (Figure 6B). PTFE_AC was fabricated following instructions from the literature [27]. The materials trialled were (a) a solid commercially available coconut shell-derived sintered carbon block filter cartridge (Water Filter Man Ltd., Tipton, UK) named AC_BLOCK (Figure 6A) and (b) a custom made PTFE-free/alginate-based electrode named PTFE_FREE_AC (Figure 6C), which was made using activated carbon (80 g) and alginate (Minerals Water Ltd., Essex, UK, 20 g). These two were mixed with distilled water into a thick paste. Prior to mixing, carbon and alginate were homogenised using an electric coffee grinder (Andrew James 150 W, Seaham, UK). The paste was then transferred to a syringe from where it was extruded directly onto the membrane (10 mL) (Figure 7) and dried/solidified on the bench in 24 h (Figure 8). This mixture was enough for 10 electrodes (2 g of alginate/electrode). All the electrodes had a projected surface area of 20 cm^2^ and the final weight of all the dried electrodes was 3.8 ± 0.2 g.

### 4.3. Operating Conditions

The cells were inoculated with acclimated activated sludge, supplied from the Wessex Water Scientific Laboratory (Saltford, UK), supplemented (1:10) with full strength (1.5% *w/v*) tryptone yeast extract (TYE) for the initial five days. TYE was used as a background solution, supplementing bacterial cells with nutrients and amino acids. The stock solution of 1.5% TYE (1% Tryptone and 0.5% Yeast Extract) was prepared using 10 g of Tryptone (Sigma Aldrich, Dorset, UK) and 5 g of Yeast Extract (Sigma Aldrich) in a litre of deionised water before it was diluted in a stock of activated sludge. The final concentration in the sludge solution was 0.15% TYE. The inoculation regime consisted of daily full exchanges (four in total). Then the MFCs were fed daily (batch mode) with neat human urine, and they were operating under a 2.7 kΩ load for the whole duration of the experiment. Urine was collected from healthy individuals, through a 125 L collection tank connected to an adapted male urinal at Bristol Robotics Laboratory (Bristol, UK). At the time of collection from the tank, the pH of urine was, on average, pH ±9.20, the conductivity was ±29.1 mS and COD was ±5.16 g/L.

### 4.4. Polarisation Experiment

Polarisation experiments were carried out by connecting the MFCs to an 8-channel automated Resistorstat [28]. The external resistance (Rext) values ranged from 30 kΩ down to 3.74 Ω with each resistance value held for 3 min. During polarisation, voltage output was recorded every 30 s (6 samples per resistance value) in order to monitor and capture the dynamic response of MFCs to changes in Rext. The MFCs were kept in open-circuit voltage for 2 h prior to polarisation testing.

### 4.5. Electrochemical Analysis of the Cathode Electrode

Linear sweep voltammetry (LSV) was performed to examine the electrocatalytic activities of the cathode electrodes under investigation. Before the electrochemical analysis took place, MFCs were left to stabilise overnight with neat urine as their anolyte solution. This allowed the membrane to be fully hydrated and guarantee liquid continuity. Cathode polarisation curves were run from open circuit voltage (OCV) to −250 mV (vs. Ag/AgCl) with a scan rate of 0.25 mV·s^−1^. LSV was performed using an SP-50 potentiostat/galvanostat from Biologic, France. For this analysis, a three-electrode configuration was used with a reference electrode (Ag/AgCl) inserted in the anodic, counter electrode’s solution. This was due to the fact that the cathode electrode was open to air, i.e., with no liquid electrolyte for the reference electrode to be inserted in. The anode was used as the counter electrode, the cathode as the working electrode and the reference channel was connected to the reference electrode placed in the anodic solution. This is contrary to the traditional use of the three-electrode setup [29] and so the data presented in Figure 4 and their analysis have taken this into consideration. For statistical significance, every electrochemical experiment was run in triplicates.

## 5. Conclusions

The key to advance MFC technology is to optimise the construction and design of units using 3D fabrication techniques. The 3D printed/extruded MFCs will not only accelerate the manufacturing process of individual units but can also help in automating the production process of multiple units for scale-up. This will benefit the electrical power output, as rapidly fabricated multiple units can be stacked together to increase voltage or current output [30,31]. Another important advantage of 3D-printing MFCs will be the standardisation of the technology. Currently, a standard MFC archetype does not exist, making the comparison of MFCs between groups around the world difficult. The possibility of 3D-printing MFCs can standardise the design, making it easily accessible and exchangeable with different research groups globally that have access to a 3D-printer.

This study demonstrated the development of a cost effective, eco-friendly, air-dried, extrudable, 3D-printed electrode (PTFE_FREE_AC) which could successfully act as a cathode electrode in an MFC system that employs air-breathing cathodes. The conductive element was initially manually deposited to evaluate the effectiveness of the material as a cathode and the experiment showed its superiority over the other two tested electrodes, PTFE_AC and AC_BLOCK. Finally, the electrode was successfully extruded from the on-board EvoBot syringe without the need for any hardware modifications to the unit. Hence, it was shown that replacing the conventional PTFE-based AC carbon electrodes with the alginate-based conductive paste is a valuable development towards printable MFCs, since both the membrane [18] and the cathode electrodes can emerge from the same platform.
